# Detection of the new SARS-CoV-2 variants of concern B.1.1.7 and B.1.351 in five SARS-CoV-2 rapid antigen tests (RATs), Germany, March 2021

**DOI:** 10.2807/1560-7917.ES.2021.26.16.2100413

**Published:** 2021-04-22

**Authors:** Sabrina Jungnick, Bernhard Hobmaier, Lena Mautner, Mona Hoyos, Maren Haase, Armin Baiker, Heidi Lahne, Ute Eberle, Clara Wimmer, Sabrina Hepner, Annika Sprenger, Carola Berger, Alexandra Dangel, Manfred Wildner, Bernhard Liebl, Nikolaus Ackermann, Andreas Sing, Volker Fingerle, the Bavarian SARS-CoV-2-Public Health Laboratory Team, Bavarian SARS-CoV-Public Health Laboratory Team, Bavarian SARS-CoV-Public Health Laboratory Team, Bernadett Bartha-Dima, Katja Bengs, Anja Berger, Kerstin Boll, Anja Carl, Christian Jürgen, Juliana Drdlicek, David Eisenberger, Jennifer Flechsler, Lars Gerdes, George Githure, Janani Govindaswamy, Christine Hupfer, Regina Konrad, Gaia Lupoli, Johannes Lutmayr, Gabriele Margos, Roswitha Müller, Silke Nickel, Mercy Okeyo, Melanie Pavlovic, Sven Pecoraro, Isabel Sahm, Melanie Schauer, Anika Schülein, Eva-Maria Schürmann, Gesine Schulze, Nelly Scuda, Stefanie Singer, Thorsten Stellberger, Bianca Treis, Christian Tuschak, Pia Zimmermann, Natali Paravinja

**Affiliations:** 1 Public Health Microbiology Unit, Bavarian Health and Food Safety Authority, Oberschleißheim, Germany; 2 These authors contributed equally to this work; 3 Unit of molecular biologic analytics and biogenetics, Bavarian Health and Food Safety Authority, Oberschleißheim, Germany; 4 Ludwig Maximilians-Universität, Munich, Germany; 5 Bavarian State Institute of Health, Oberschleißheim, Germany; 6 Members of the Bavarian SARS-CoV-Public Health Laboratory Team are listed below

**Keywords:** SARS-CoV-2, Rapid antigen test(s), Variant(s) of Concern, performance, B.1.1.7, B.1.351

## Abstract

SARS-CoV-2 variants of concern (VOC) should not escape molecular surveillance. We investigated if SARS-CoV-2 rapid antigen tests (RATs) could detect B.1.1.7 and B.1.351 VOCs in certain laboratory conditions. Infectious cell culture supernatants containing B.1.1.7, B.1.351 or non-VOC SARS-CoV-2 were respectively diluted both in DMEM and saliva. Dilutions were analysed with Roche, Siemens, Abbott, nal von minden and RapiGEN RATs. While further studies with appropriate real-life clinical samples are warranted, all RATs detected B.1.1.7 and B.1.351, generally comparable to non-VOC strain.

Since the first report of a severe acute respiratory syndrome coronavirus 2 (SARS-CoV-2) variant of concern (VOC) in December 2020, other VOCs have emerged, and the question as to what extent these could influence the performance of SARS-CoV-2 rapid antigen tests (RATs) has arisen [1-3]. Therefore, as soon as the first B.1.1.7 and B.1.351 VOC isolates were obtained at the Bavarian Health and Food Safety Authority in Germany, these were used to find out if various RATs could detect such variants.

## Rapid antigen tests studied

The following RATs were chosen for further study: (i) Roche – SARS-CoV-2 Rapid Antigen Test (further referred to as test I; lot QCO3020083/SubA1, expiration date (Exp.) 15 Sep 2022; Roche, Mannheim, Germany), (ii) Siemens – CLINITEST Rapid COVID-19 Antigen Test (test II; lot 2011317, Exp. 31 Oct 2022; Siemens, Erlangen, Germany), (iii) Abbott – Panbio COVID-19 Ag RAPID TEST DEVICE (test III; lot 41ADF363A/SubA, Exp. 25 Nov 2021; Abbott, Jena, Germany), (iv) nal von minden – NADAL COVID-19 Ag rapid test (test IV; lot 175009, Exp. Aug 2022; nal von minden, Moers, Germany) and (v) RapiGEN – BIOCREDIT COVID-19 Ag rapid test kit (test V; lot H073009SD, Exp. 05/31/2022; RapiGEN, Anyang, South Korea).

## Preparation of laboratory samples for rapid antigen testing

As this was not a clinical validation study, investigating whether the RATs could detect VOCs, was conducted based on an in vitro laboratory approach. No real-life clinical samples were used and test samples were instead generated using infectious SARS-CoV-2 viruses derived from cell culture. For this, isolation and cultivation of SARS-CoV-2 clinical isolates were performed as described previously [4]. Vero E6 cell culture supernatants containing infectious SARS-CoV-2 were collected 72 hours post infection, aliquoted and stored at − 80 °C until further use.

Infectious cell culture supernatants of the VOCs B.1.1.7 and B.1.351 (both confirmed by whole genome sequencing (WGS) from the passage before final expansion), as well as of a non-VOC SARS-CoV-2 isolate were used to test the RATs. The non-VOC SARS-CoV-2 virus had been isolated in March 2020 and tested negative for mutation N501Y, by variant-specific reverse transcription quantitative (RT-q)PCR (ViRSNiP assay, TIB MOLBIOL, Berlin, Germany).

## Limit of detection for cell-culture derived SARS-CoV-2 viruses

To determine the analytical limit of detection (LoD) for the cell culture supernatants with infectious SARS-CoV-2 viruses, we prepared dilution series in both Dulbecco’s modified Eagle’s medium (DMEM) and in pooled saliva from voluntary, asymptomatic donors, with negative results in SARS-CoV-2 RT-qPCR (PCR-protocol described below). Saliva was used in addition to DMEM as a carrier material, to mimic conditions in biological material. It should be noted, however, that saliva is not the sample type recommended by the RATs’ manufacturers.

Respective RAT extraction buffers were inoculated with 50 µL of each dilution, before following the manufacturer’s instructions for further testing. For each RAT, every dilution was tested in three parallel runs. The LoD was determined as the dilution level at which two investigators could still clearly visually identify positive test strips in all triplicates independently and blinded with regard to concentration levels. In case of ambiguity, a third investigator was consulted (reference bands for evaluations shown in the Figure). 

**Figure:**
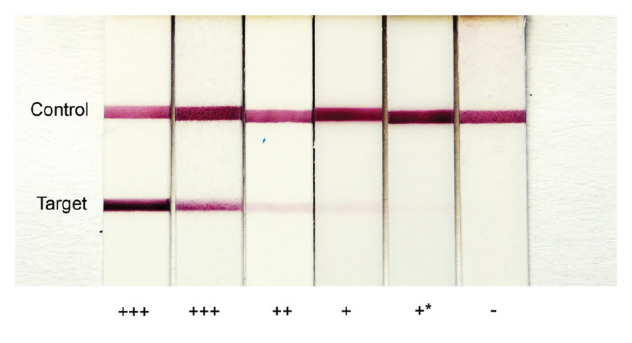
Reference bands for evaluation and grading of rapid antigen test results, March 2021

Examples, from one type of rapid antigen test, of results that were considered strong positive ( +  +  + ), weak positive ( +  + ), very weak positive ( + ), as well as very weak positive and not clearly detectable in all tests or by all investigators ( + *); a negative result (−) is also shown. The target band visual intensity is used to determine the result’s positivity strength when the control band, which indicates that the test is working, is present. The first two strips on the left are both ‘+  +  +’ and are both shown to give a visual range of outputs considered strongly positive.

After being stored for 24 hours at 4 °C, all dilution levels, including negative controls for DMEM and saliva, were heat inactivated (65 °C for 90 min) and tested in parallel by RT-qPCR as described previously [5]. Briefly, the RNAdvance Viral Large GRP Kit (Beckmann-Coulter Life Sciences, Nyon, Switzerland) was used to extract viral RNA on a Microlab STAR (Hamilton Company, Reno, United States (US)). The RNA was subjected to RT-qPCR with the AmpliCube Coronavirus SARS-CoV-2 RT-qPCR Kit (Mikrogen, Neuried, Germany) on the Bio-Rad CFX96 Real-Time RT-qPCR Detection System (Bio-Rad, Feldkirchen, Germany) [5]. For quantification, the EDX SARS-CoV-2 Standard (Exact diagnostics, Fort Worth, Texas, US) was used as a reference RNA-standard. 

Furthermore, infectivity of the used supernatants (50-per cent tissue culture infective dose; TCID50/mL) was determined via endpoint dilution assay.

## Ethical statement

No ethical approval was necessary for this study, as no clinical specimens from patients were used.

## Rapid antigen test results on SARS-CoV variants

Irrespective of whether the virus was a SARS-CoV-2 VOC or not, we generally noticed, in line with previous independent studies [6,7], differences in the LoD of RATs depending on the manufacturer (Table 1). Tests I–IV showed an overall comparable performance and were mostly able to detect the antigen in 1:1,000 dilutions (RNA copies/mL approx. 8.5 × 10^5^–9.8 × 10^5^ (DMEM) and 1.1 × 10^6^–1.9 × 10^6^ (saliva)). In contrast, test V required notably higher virus concentrations (1:10 dilution, RNA copies/mL approx. 2.6 × 10^7^–3.4 × 10^7^ (DMEM) and 3.9 × 10^7^–6.6 × 10^7^ (saliva)) to detect SARS-CoV-2 in cell culture supernatants.

**TABLE 1:** Determination of the limit of detection of various rapid antigen tests for SARS-CoV-2 and variants of concern, based on virus dilution series in Dulbecco’s modified Eagle’s medium and saliva, March 2021

Test number^a^	Virus characteristic	Dilution					LoD in RNA copies/mL^b^
		1:10	1:100	1:1,000	1:10,000	1:100,000	
**Dilution in DMEM**							
**Test I**	Non-VOC	ND	+ + +	+ + +	Neg.	Neg.	9.8 × 10^5^
	B.1.351	ND	+ + +	+ + +	Neg.	Neg.	8.5 × 10^5^
	B.1.1.7	ND	+ + +	+ + +	Neg.	Neg.	8.9 × 10^5^
**Test II**	Non-VOC	ND	+ + +	+ +	+	Neg.	1.7 × 10^5^
	B.1.351	ND	+ + +	+ +	+^c^	Neg.	8.5 × 10^5^
	B.1.1.7	ND	+ + +	+ +	Neg.	Neg.	8.9 × 10^5^
**Test III**	Non-VOC	ND	+ + +	+ + +	+^d^	Neg.	9.8 × 10^5^
	B.1.351	ND	+ + +	+ + +	+^d^	Neg.	8.5 × 10^5^
	B.1.1.7	ND	+ + +	+ + +	Neg.	Neg.	8.9 × 10^5^
**Test IV**	Non-VOC	ND	+ + +	+ +	Neg.	Neg.	9.8 × 10^5^
	B.1.351	ND	+ + +	+ +	Neg.	Neg.	8.5 × 10^5^
	B.1.1.7	ND	+ + +	+ +	Neg.	Neg.	8.9 × 10^5^
**Test V**	Non-VOC	+ + +	Neg.	Neg.	Neg.	Neg.	3.1 × 10^7^
	B.1.351	+	Neg.	Neg.	Neg.	Neg.	2.6 × 10^7^
	B.1.1.7	+ +	Neg.	Neg.	Neg.	Neg.	3.4 × 10^7^
**Dilution in saliva**							
**Test I**	Non-VOC	ND	+ + +	+ +	Neg.	Neg.	1.3 × 10^6^
	B.1.351	ND	+ + +	+ +	Neg.	Neg.	1.1 × 10^6^
	B.1.1.7	ND	+ + +	+ +	Neg.	Neg.	1.9 × 10^6^
**Test II**	Non-VOC	ND	+ + +	+ +	+	Neg.	2.2 × 10^5^
	B.1.351	ND	+ + +	+ +	+^c^	Neg.	1.1 × 10^6^
	B.1.1.7	ND	+ + +	+ +	Neg.	Neg.	1.9 × 10^6^
**Test III**	Non-VOC	ND	+ + +	+ +	Neg.	Neg.	1.3 × 10^6^
	B.1.351	ND	+ + +	+ +	Neg.	Neg.	1.1 × 10^6^
	B.1.1.7	ND	+ + +	+ +	Neg.	Neg.	1.9 × 10^6^
**Test IV**	Non-VOC	ND	+ + +	+ +	Neg.	Neg.	1.3 × 10^6^
	B.1.351	ND	+ +	Neg.	Neg.	Neg.	6.4 × 10^6^
	B.1.1.7	ND	+ + +	+ +	Neg.	Neg.	1.9 × 10^6^
**Test V**	Non-VOC	+ + +	Neg.	Neg.	Neg.	Neg.	4.7 × 10^7^
	B.1.351	+	Neg.	Neg.	Neg.	Neg.	3.9 × 10^7^
	B.1.1.7	+ +	Neg.	Neg.	Neg.	Neg.	6.6 × 10^7^

DMEM: Dulbecco’s modified Eagle’s medium; LoD: limit of detection; ND: not determined; neg: negative; SARS-CoV-2: severe acute respiratory syndrome coronavirus; VOC: variant of concern.

The test results are described as very weak positive (+), weak positive (+  +), or strong positive (+  +  +).

^a^ Test I: Roche – SARS-CoV-2 Rapid Antigen Test; test II: Siemens – CLINITEST Rapid COVID-19 Antigen Test; test III: Abbott – Panbio COVID-19 Ag RAPID TEST DEVICE; test IV: nal von minden – NADAL COVID-19 Ag rapid test; test V: RapiGEN – BIOCREDIT COVID-19 Ag rapid test kit.

^b^ Cells shaded in grey-blue colour indicate the dilution determining the LoD.

^c^ Only two of three investigators could clearly identify a positive target band.

^d^ Only one of three replicates showed a very faint target band.

Regarding the test results for the non-VOC isolate vs the VOCs B.1.1.7 and B.1.351, the LoD was identical for all three variants in tests I, III, and V. Test II showed a lower LoD for the non-VOC isolate in both DMEM and saliva, detecting the antigen in 1:10,000 diluted supernatants in comparison to a LoD at 1:1,000 dilutions for B.1.1.7 and B.1.351. Test IV detected all variants equally in DMEM at 1:1,000 dilutions, while the LoD of the B.1.351 variant in saliva was at a 1:100 dilution. Small differences within the initial concentrations are to be mentioned (Table 2).

**TABLE 2:** Corresponding reference values for all dilution steps based on RT-qPCR, March 2021

Samples		Dilution in DMEM			Dilution in saliva			
		Non-VOC	B.1.351	B.1.1.7	Non-VOC	B.1.351	B.1.1.7	
**Initial preparation**	**Viral concentration** **(RNA copies/mL)**	**3.8 × 10^8^**	**3.1 × 10^8^**	**4.8 × 10^8^**	**NA**			
	**Infectivity (TCID50/mL)**	**3.84 × 10^5^**	**1.49 × 10^5^**	**3.45 × 10^5^**				
	**Ct value *Orf1* gene**	**12.94**	**13.30**	**12.48**				
**1:10**	Viral concentration (RNA copies/mL)	3.1 × 10^7^	2.6 × 10^7^	3.4 × 10^7^	4.7 × 10^7^	3.9 × 10^7^		6.6 × 10^7^
	RNA copies per 50 µL inoculum	1.6 × 10^6^	1.3 × 10^6^	1.7 × 10^6^	2.4 × 10^6^	2.0 × 10^6^		3.3 × 10^6^
	Ct value *Orf1* gene	17.76	18.10	17.58	16.95	17.32		16.33
**1:100**	Viral concentration (RNA copies/mL)	5.6 × 10^6^	4.5 × 10^6^	5.5 × 10^6^	7.8 × 10^6^	6.4 × 10^6^		9.6 × 10^6^
	RNA copies per 50 µL inoculum	2.8 × 10^5^	2.3 × 10^5^	2.7 × 10^5^	3.9 × 10^5^	3.2 × 10^5^		4.8 × 10^5^
	Ct value *Orf1* gene	21.10	21.52	21.15	20.46	20.86		20.07
**1:1,000**	Viral concentration (RNA copies/mL)	9.8 × 10^5^	8.5 × 10^5^	8.9 × 10^5^	1.3 × 10^6^	1.1 × 10^6^		1.9 × 10^6^
	RNA copies per 50 µL inoculum	4.9 × 10^4^	4.2 × 10^4^	4.4 × 10^4^	6.7 × 10^4^	5.5 × 10^4^		9.3 × 10^4^
	Ct value *Orf1* gene	24.49	24.77	24.68	23.90	24.25		23.24
**1:10,000**	Viral concentration (RNA copies/mL)	1.7 × 10^5^	1.5 × 10^5^	1.7 × 10^5^	2.2 × 10^5^	2.0 × 10^5^		1.7 × 10^5^
	RNA copies per 50 µL inoculum	8.3 × 10^3^	7.6 × 10^3^	8.4 × 10^3^	1.1 × 10^4^	1.0 × 10^4^		8.7 × 10^4^
	Ct value *Orf1* gene	27.94	28.11	27.92	27.38	27.55		27.85
**1:100,000**	Viral concentration (RNA copies/mL)	3.0 × 10^4^	2.6 × 10^4^	4.2 × 10^4^	3.6 × 10^4^	3.7 × 10^4^		4.0 × 10^4^
	RNA copies per 50 µL inoculum	1.5 × 10^3^	1.3 × 10^3^	2.0 × 10^3^	1.8 × 10^3^	1.9 × 10^3^		2.0 × 10^3^
	Ct value *Orf1* gene	31.25	31.52	30.63	30.90	30.85		30.70

Ct: cycle threshold; DMEM: Dulbecco’s modified Eagle’s medium; NA: not available; Orf1: open reading frame 1; RT-qPCR: reverse transcription-quantitative PCR; TCID50: 50-per cent tissue culture infective dose.

Quantification of RNA copy numbers via reference SARS-CoV-2 standard (*Orf1* gene). TCID50/mL determined by endpoint dilution assay.

## Discussion

Since SARS-CoV-2 RATs were first approved by the US Food and Drug Administration (FDA) in May 2020, they became an integral part of international SARS-CoV-2 containment strategies [8,9]. As SARS-CoV-2 VOCs spread worldwide, a verification of RAT performance with these VOCs is mandatory [2,10,11]. All RATs evaluated in this study, as well as most SARS-CoV-2 RATs currently available, are based on detection of the nucleocapsid protein (N-protein). Mutations like N501Y (B.1.1.7/B.1.351) and the H69/V70 deletion (B.1.1.7), however, result in alterations of the spike protein (S-protein), which is currently less frequently used by RATs [11]. Although a general focus regarding VOCs is laid on mutations in the spike protein, there are also numerous mutations in the N-protein (e.g. N:D3L and N:S235F in B.1.1.7 and N:T205I in B.1.351), which may in some instances alter its dynamic stability and immunogenic properties [12,13]. Such N-protein mutations, which are already present or may emerge during the course of the pandemic, could influence the test performance of N-specific SARS-CoV-2 RATs. Thus, a continuous evaluation of SARS-CoV-2 RAT performance is obligatory, especially with regard to evolving mutations. According to the global health non-profit Foundation for Innovative New Diagnostics (FIND), an assessment by Public Health England found that the variant B.1.1.7 was equally well detected by all of five evaluated SARS-CoV-2 RATs (including Abbott Panbio, Fortress, Innova, Roche/SD Biosensor nasal swab, and Surescreen). However, according to FIND, comparable studies have not yet been conducted for B.1.351 (status at 1 April 2021) [11,14]. Therefore, further independent investigations – like this rapid report – are deemed necessary.

Some limitations of the current study should nevertheless be emphasized. The assessment of whether RATs could detect VOCs was laboratory-based and no real-life clinical samples were tested. As such, the study should not be considered a clinical validation study but an in vitro laboratory analysis aiming at analytical performance data. To mimic conditions in biological material, saliva was used as a carrier material in addition to DMEM (the medium used for cell culture), but all five RATs employed are currently not approved for use with saliva by their manufacturers. However, no distinct performance differences could be determined between saliva and DMEM. Another limitation is that in order to generate test samples for assessing the RATs, viruses in cell-culture medium, which were obtained after passage on Vero cells, were used. While VOC and non-VOC viruses were verified by WGS or variant-specific RT-qPCR using the passage before final expansion, mutations could have occurred in the viruses during the final passage, affecting the results. Moreover, all RATs investigated are meant for manual reading of results. Therefore, despite standardised evaluation conditions, no quantitative result evaluation could be offered. It should be nonetheless noted that these evaluation conditions reflect the conditions present in the clinical and practical application of those tests.

## Conclusion

Our results show all RATs investigated were able to detect the variants B.1.1.7 and B.1.351. In general, detection of B.1.1.7 and B.1.351 by RATs in our study was comparable to that of the non-VOC strain, however, a potential impact of the SARS-CoV-2 VOCs B.1.1.7 and B.1.351 on the laboratory performance of evaluated RATs, compared with non-VOC isolates, cannot be ruled out. Therefore, we would like to emphasize that a potential influence of VOCs on test performance of RATs must be investigated individually for each RAT and each VOC, assuring their suitability as ‘COVID-19 filters’ [15]. Further studies with appropriate real-life clinical samples are also warranted.
