# Addendum for Euro Surveill. 2021;26(16)

**DOI:** 10.2807/1560-7917.ES.2021.26.24.2106171a

**Published:** 2021-06-17

**Authors:** Eurosurveillance editorial team

**Affiliations:** 1 European Centre for Disease Prevention and Control (ECDC), Stockholm, Sweden

In the article ‘Detection of the new SARS-CoV-2 variants of concern B.1.1.7 and B.1.351 in five SARS-CoV-2 rapid antigen tests (RATs), Germany, March 2021’ by S Jungnick et al., published on 22 April 2021, GenBank accession numbers were added on 17 June 2021.

