# Addendum for Euro Surveill. 2021;26(16)

**DOI:** 10.2807/1560-7917.ES.2021.26.43.211028a

**Published:** 2021-10-28

**Authors:** Eurosurveillance editorial team

**Affiliations:** 1 European Centre for Disease Prevention and Control (ECDC), Stockholm, Sweden

In the article ‘Detection of the new SARS-CoV-2 variants of concern B.1.1.7 and B.1.351 in five SARS-CoV-2 rapid antigen tests (RATs), Germany, March 2021’ by Jungnick et al. published on 22 April 2021, GISAID IDs were added and the text was changed to note that whole genome sequencing (WGS) was performed after final passage to exclude mutations. These additions were made on 28 October 2021.

